# Structural and transport properties of ammonia along the principal Hugoniot

**DOI:** 10.1038/s41598-017-12429-w

**Published:** 2017-09-26

**Authors:** Dafang Li, Cong Wang, Jun Yan, Zhen-Guo Fu, Ping Zhang

**Affiliations:** 1 Institute of Applied Physics and Computational Mathematics, Beijing, 100088 People’s Republic of China; 2 Center for Applied Physics and Technology, Peking University, Beijing, 100871 People’s Republic of China

## Abstract

We investigate, via quantum molecular dynamics simulations, the structural and transport properties of ammonia along the principal Hugoniot for temperatures up to 10 eV and densities up to 2.6 g/cm^3^. With the analysis of the molecular dynamics trajectories by use of the bond auto-correlation function, we identify three distinct pressure-temperature regions for local chemical structures of ammonia. We derive the diffusivity and viscosity of strong correlated ammonia with high accuracy through fitting the velocity and stress-tensor autocorrelation functions with complex functional form which includes structures and multiple time scales. The statistical error of the transport properties is estimated. It is shown that the diffusivity and viscosity behave in a distinctly different manner at these three regimes and thus present complex features. In the molecular fluid regime, the hydrogen atoms have almost the similar diffusivity as nitrogen and the viscosity is dominated by the kinetic contribution. When entering into the mixture regime, the transport behavior of the system remarkably changes due to the stronger ionic coupling, and the viscosity is determined to decrease gradually and achieve minimum at about 2.0 g/cm^3^ on the Hugoniot. In the plasma regime, the hydrogen atoms diffuse at least twice as fast as the nitrogen atoms.

## Introduction

As ammonia is one of the major constituent of the “hot ice” layers in Uranus and Neptune^1,2^, its properties such as diffusivity, viscosity and structure have considerable impact on the internal evolution and magnetic field of these giant planets. For example, the transport character of warm dense ammonia is essential for understanding the composition of giant planets, and also the magnetohydrodynamic models of these planets depend on the knowledge of diffusion and viscosity of the fluid^3^. In this weakly hydrogen-bonded liquid, chemical processes are expected to take place at deep planetary conditions (several 100 GPa and several 1000 K), which could induce the change of structure and transport properties of ammonia. However, the chemistry and transport properties of ammonia at extreme conditions still remain elusive up to date.

Much of the experimental and theoretical research have advanced the determination and understanding of physical properties of ammonia under extreme conditions. On experimental side, some dynamic techniques, such as standard explosive technique and two-stage light-gas gun, have been used to derive the Hugoniot of ammonia up to 39 GPa first, and later to a higher pressure of 64 GPa^4,5^. Furthermore, Radousky *et al*. measured the shock temperature at the pressures of 48 and 61 GPa for diagnosing the physics occurring in ammonia^6^. Nonmetal-to-Metal transition for ammonia was found in the electrical conductivity measurements^7,8^. Recently, the phase diagram up to 60 GPa and 2500 K has been studied by several static experiments with diamond anvil cells^9–12^. However, experiments were restricted to certain areas of the phase diagram due to the limitations of technique. More detailed and broad information at extreme conditions come from theoretical calculations. *Ab initio* molecular dynamics simulations have predicted the phase transition to a protonic conductor for ammonia above 60 GPa and 1200 K^13^. First-principles calculations have shown that ammonia molecules chemically dissociate to N_2_ and H_2_ above approximately 7 GPa and 900 K, which was thought to be driven by the entropy of mixing term in the free energy formulation^11^. In addition, *ab initio* molecular dynamics simulations have been used to examine the equation of state (EOS) and phase diagram covering pressures up to 330 GPa and a temperature range from 500 K to 10000 K^14^. We have recently investigated the thermophysical properties of shocked liquid ammonia up to the pressure of 1.3 TPa and temperature 120000 K using quantum molecular dynamics simulations. It was found that the liquid ammonia undergoes three structural regimes along the principal Hugoniot, accompanying the transformation to a metallic state^15^. Later, Mulford *et al*. predicted the ammonia EOS based on Cheetah code^16^.

In this paper, we study the structure and transport properties of shocked ammonia under extensive pressure-temperature regimes with temperatures up to 10 eV and densities 2.6 g/cm^3^ systematically. Specially, the structural transformation is first studied by the pair distribution function and bond auto-correlation function (BACF), and then the changes in the diffusion coefficients and viscosity of the system at different structural regimes are discussed in detail.

## Results

To confirm the internal energy and pressure achieve good convergence, we first conduct convergence tests for the particle number, the plane-wave cutoff and *k*-point Brillouin sampling at several *P* − *T* conditions, which include (i) QMD simulations with 64-molecule supercells, the Γ point and a 1000 eV plane-wave cutoff;  (ii) QMD simulations with 64-molecule supercells, the Γ point and a 550 eV plane-wave cutoff; (iii) QMD simulations with 27-molecule supercells, the Γ point and a 550 eV plane-wave cutoff; (iv) QMD simulations with 27-molecule supercells, a 2 × 2 × 2 *k*-point grid and a 550 eV plane-wave cutoff. We take the Hugoniot point of 2.0 g/cm^3^ and 6036 K as an example, and present the comparison results in Table 1. It can be seen that the internal energies and pressures are converged to less than 1% for all of these choices of parameters.

**Table 1:** The pressure and internal energy at the Hugoniot point of 2.0 g/cm^3^ and 6036 K for different particle number (*N*), the plane-wave cutoff (*E* cut), *k*-point Brillouin sampling and exchange-correlation potentials (XC).

*N*	*E*_cut_ (eV)	*K*-point	XC	*P* (GPa)	*E* (kJ/g)
64	1000	1 × 1 × 1	PW91	96.48	−60.164
64	550	1 × 1 × 1	PW91	96.11	−60.258
27	550	1 × 1 × 1	PW91	95.94	−60.559
27	550	2 × 2 × 2	PW91	96.21	−60.330
27	550	1 × 1 × 1	PBE	95.76	−59.846
27	550	1 × 1 × 1	LDA	86.42	−69.986

**Figure 1:**
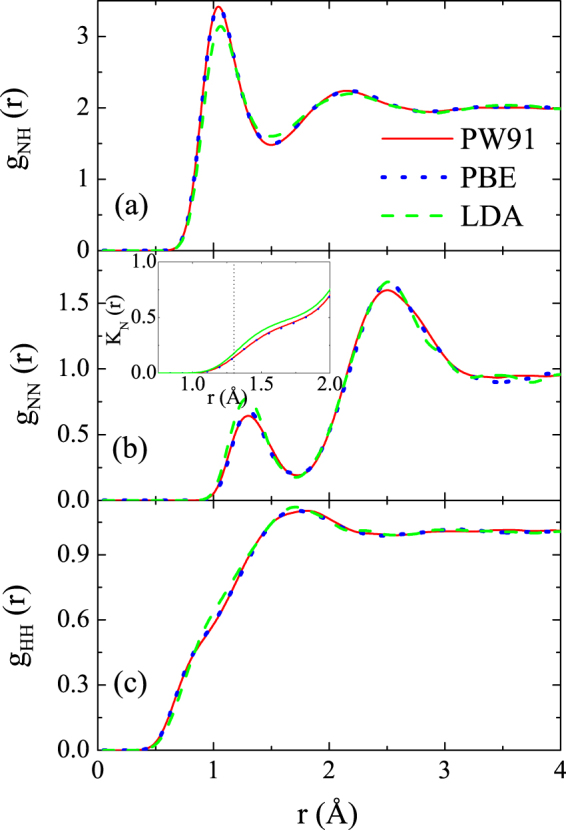
Pair distribution function for three types of bonds of (**a**) N-N, (**b**) H-H, and (**c**) N-H for three different exchange-correlation potential (PW91, PBE, and LDA) at Hugoniot point of 2.0 g/cm^3^ and 6036 K. The inset is the coordination number of N.

We further examine the influence of the exchange-correlation potential (PW91, PBE, and LDA) on the equations of state. In Table 1, the pressures and internal energies for different exchange-correlation potentials are also included. The data from QMD simulations with PW91 functional show good agreement with those from PBE functional, while the data from LDA functional present prominent discrepancies, with the relative deviations between PW91 and LDA results as large as 9.9% and 15.6% for pressure and internal energy, respectively. The large deviations may arise from the inaccurate modeling of the breaking and forming of chemical bonds for LDA functional. And thus we present the impact of the exchange-correlation potential on the pair distribution functions (PDFs) in Fig. 1. The PW91 and PBE functionals yield almost the same PDFs, while the LDA functional predicts reduced peak in g_NH_(*r*) and higher peak in g_NN_(*r*), which signify the larger fraction of dissociated ammonia molecules and formation of N-N bonding. Furthermore, we look insight into the N-N bonding in ammonia through calculating the coordination numbers of N, which is a weighted integral over the PDFs g(*r*) of N-N $(K(r)=\frac{N-1}{V}\,{\int }_{0}^{r}\,4\pi {r}^{^{\prime} 2}g\,(r^{\prime} )\,{\rm{d}}r^{\prime} )$. The fraction of the ions bounded to a nitrogen molecule is twice the value of *K*(*r*) at the first peak in *g*(*r*), which is found at around 1.30Å. The plots are inset into Fig. 1(b). The coordination number of N is 0.136 for the PW91 and PBE functionals, while 0.175 for the LDA functional. Thus the fraction of N-N bonding is 27.2% and 35% for PW91/PBE functional and LDA functional, respectively.

**Figure 2:**
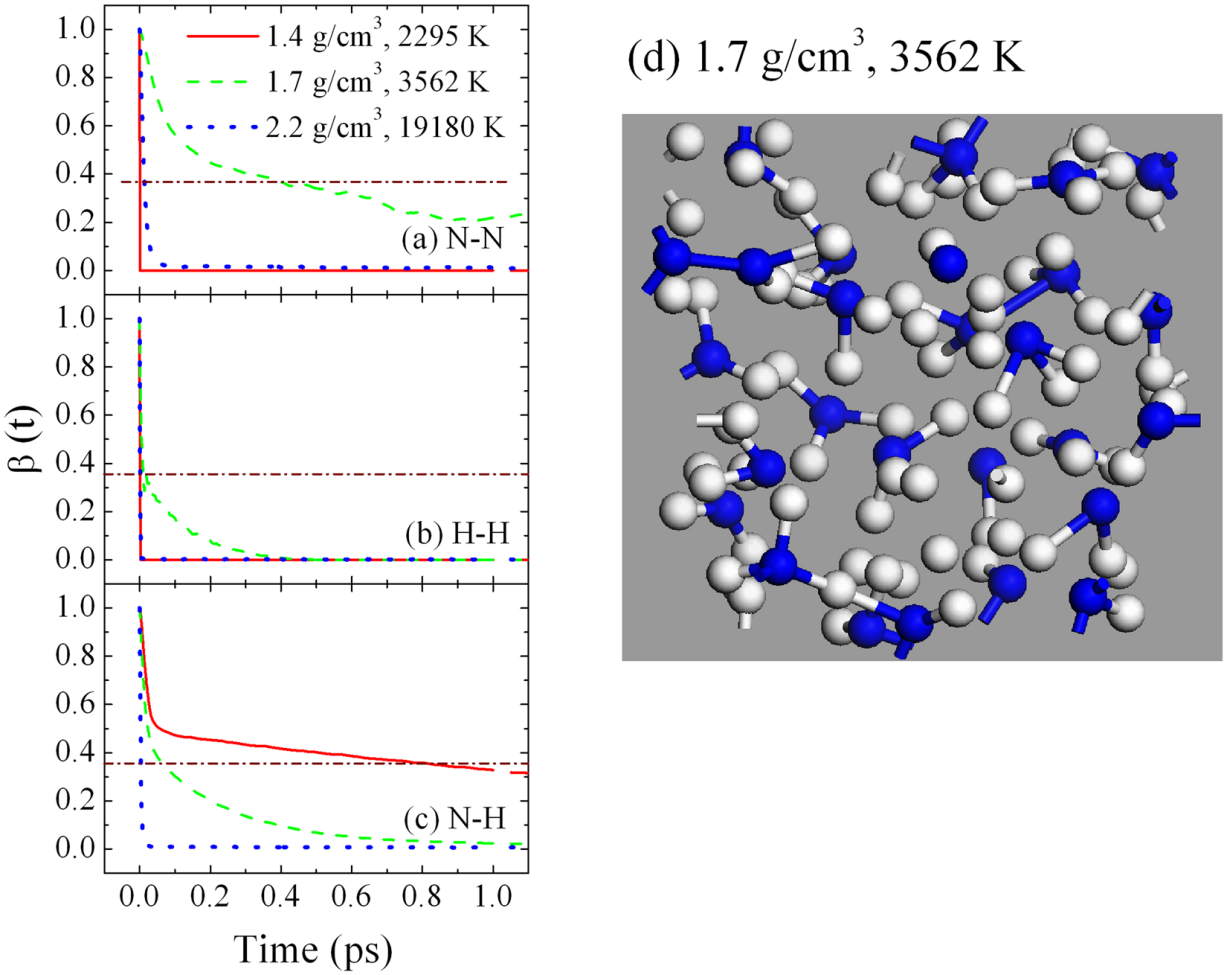
BACF for three types of bonds: (**a**) N-N, (**b**) H-H, and (**c**) N-H for three different Hugoniot points; (**d**) A snapshot of the MD simulation at density of 1.7 g/cm^3^ at 3562 K. Nitrogen atoms are blue or grey balls, and hydrogen atoms are light grey balls. The horizontal dashed line represents the value of 1/*e*.

In the following, we examine the chemistry that occurs in ammonia along the principal Hugoniot. The Hugoniot points have been obtained using QMD simulations in our previous studies^15^. Figure 2 presents the BACF of ammonia for three types of bonds: N-N, N-H and H-H for different Hugoniot points. At 1.4 g/cm^3^ and 2295 K, the bond lifetimes for N-N and H-H are very short, even close to zero. While N-H bonds are at least an order of magnitude longer. This demonstrates that the system mainly remains as ammonia molecules at lower densities and temperatures of the Hugniot. At the intermediate density and temperature, the bond lifetime for N-H becomes much shorter, smaller than 0.2 ps (time for which the correlation function is equal to 1/*e*). N-N bonds last about six times longer. It is indicated that ammonia molecules dissociate and small amounts of nitrogen bonds form at this state. The mixture phase could also be seen more obviously from the snapshot of the MD simulation of 1.7 g/cm^3^ at 3562 K shown in Fig. 1(d). With increasing the density and temperature to 2.2 g/cm^3^ and 19180 K, respectively, all three types of molecules are very short-lived and unstable, with the system entering into a plasma state.

**Figure 3:**
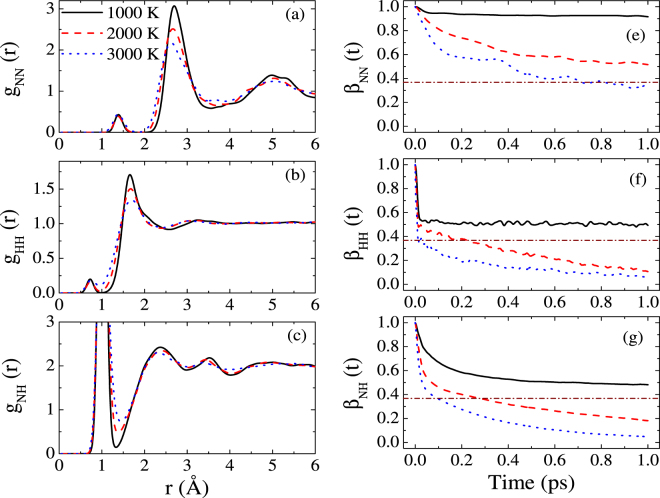
Pair distribution function of (**a**) N-N, (**b**) H-H and (**c**) N-H; BACF for three types of bonds: (**e**) N-N, (**f**) H-H, and (**g**) N-H along the isochore of 1.8 g/cm^3^ for three different temperatures.

In order to gain a further insight into the structural properties of ammonia in the mixture region, we present in Fig. 3 the PDF and BACF for three types of bonds along an isochore of 1.8 g/cm^3^ at different temperatures. For the nitrogen-nitrogen PDF, the first peak is at the position of 1.38 Å, longer than the length of triple bond. This signifies the formation of the chain of nitrogen atoms. Here the expression of “the chain of nitrogen atoms” denotes the formation of single bond of N-N. Based on the bond-length criteria, the N-N bonding could be identified. We have used a cutoff radius of 1.70 Å, which is the maximum value for single N-N bond length, to construct a sphere about each nitrogen and the bonding between two atoms is defined as a series of overlapping spheres. It is found that at the corresponding states of 1.8 g/cm^3^ and 1000/2000/3000 K, only the nitrogen chains with the typical length of two occur. In addition, we have calculated the fractions of N-N bond as 11.6%, 11.6% and 16% for these three states, respectively. Meanwhile, quite a few hydrogen molecules appear. In addition, the first minimal value of g_NH_(*r*) is slightly larger than zero, indicating the occurrence of dissociations of ammonia molecules. At lower temperature, the lifetimes for all three types of bonds are extremely long, indicative of the mixture state of ammonia. With increasing temperature, ammonia dissociates continuously and give rise to shorter lifetime for N-H bond.

As we have reported in ref.^15^, the principal Hugoniot of ammonia presents three characterized segmentations, which results from the structural changes. And thus we further explore the effect of structural changes on the diffusion and viscosity properties. Based on QMD simulations, we derive the time-dependent diffusion and viscosity coefficients of ammonia using Eqs (2–4) and (6). It should be noted that the mutual diffusion coefficients are calculated with the thermodynamic factor estimated by (〈Z*〉 + 〈(Z*)^2^〉)/(〈Z*〉 + 〈Z*〉^2^). Numerical calculations based on average-atom model are performed to determine the average ion Z*^17^. We tabulate the thermodynamic factor for each Hugoniot point in Table 2. As expected, this factor is larger than one, due to an ambipolar effect, depending on the symmetry of the mixture. Through fitting the QMD results using the functional formula of Eqs (7), (9) and (11), the self- and mutual-diffusion coefficients, and viscosity are obtained with the correlation time between 5 to 100 fs. A total uncertainty less than 20% in mutual diffusion coefficient and viscosity come from the fitting and extrapolation to infinite time, while the error in the self diffusion coefficient is less than 4% since the particle average gives an additional $\frac{1}{\sqrt{N}}$ factor.

**Table 2:** The thermodynamic factor for mutual diffusion coefficient along the principal Hugoniot of ammonia.

*ρ* (g/cm^*m*^)	0.8	1.0	1.2	1.4	1.6	1.7	1.8	1.9	2.0	2.2	2.4	2.6
*T* (K)	250	710	1735	2295	3562	3082	4174	4791	6036	19180	54229	112663
*Q*	1.151	1.189	1.221	1.247	1.270	1.280	1.289	1.298	1.306	1.322	1.334	1.353

**Figure 4:**
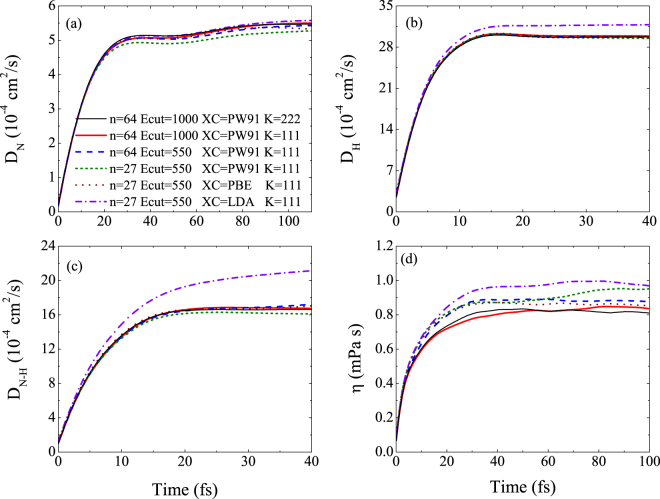
(**a**) Self-diffusion coefficient of N, (**b**) self-diffusion coefficient of H, (**c**) mutual-diffusion coefficients, and (**d**) viscosity of ammonia for different particle number, energy cutoff and exchange-correlation potential at Hugoniot point of 2.0 g/cm^3^ and 6036 K.

We examine the convergence of the transport properties with respect to particle number, energy cutoff, *k*-points and exchange-correlation potential, as shown in Fig. 4. It can be seen that for different particle numbers and energy cutoff, both the self-diffusion coefficient of N and H are converged well, while for mutual-diffusion coefficient and viscosity, smaller particle number and energy may yields about 7.3% and 10.7% error, respectively. Thus in the whole simulations of this paper we set the particle number as 64 and energy cutoff as 1000 eV. For the different K point sets of ‘111’ and ‘222’, all of the self and mutual diffusion coefficients converged very well, while the viscosity deviates a little, but still less than 5%. As for the impact of exchange-correlation potential on these transport coefficients, the PW91 functional and PBE functional give the very similar results, while the LDA functional diverge from them.

**Figure 5:**
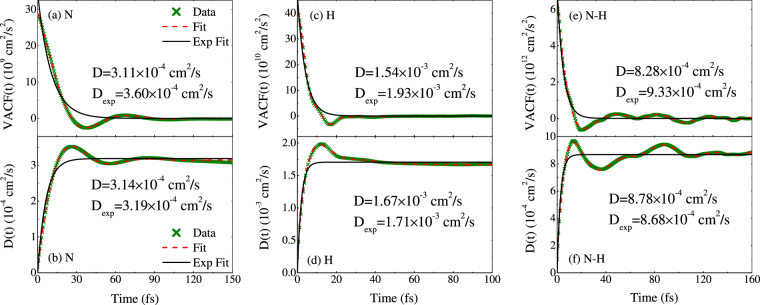
The data from QMD run on ammonia at 1.9 g/cm^3^ and 4791 K. (**a**) VACF for N in ammonia using Eq. (7) with *i* = 1; (**b**) Self-diffusion coefficient for N in ammonia using the fit to the analytic integral of Eq. (7); (**c**) VACF for H in ammonia using Eq. (7) with *i* = 2; (**d**) Self-diffusion coefficient for H in ammonia using the fit to the analytic integral of Eq. (7); (**e**) VACF for N-H using Eq. (9) with *i* = 3; (**d**) Mutual-diffusion coefficient for N-H using the fit to the analytic integral of Eq. (9). The green “x” are the numerical data, while the light-red dashed line is the fits using the respective equations. The solid black line is the example of a single exponential fit.

In Fig. 5, we show the fits to the velocity autocorrelation function (VACF) for ammonia at 1.9 g/cm^3^ and 4791 K as well as the numerically integrated results. It is evident that correlated behavior and structures exist in these three kinds of VACF. The functional of Eqs (7) and (9) and their analytic integrals fit the raw data well. The diffusion coefficients coming from the fit to the VACF and its analytic integral are in statistical agreement with each other using the standard error in Eq. (13). We further find that there are different time scales and structures in N, H and N-H. In the fit for N, one type of restoring force could give a better fit via the ${\bar{R}}^{2}$ criteria, while for H, a secondary restoring force is required, and even for N-H, all of the forces of N-N, H-H, and N-H are needed. The fit using a single exponential are also included (indicated as the solid black line). The single exponential cannot describe the oscillating behavior of the VACF and its integral, but goes to zero quickly. Thus the fitting to VACF using the single exponential yield higher diffusion coefficients than that of the fit using Eq. (7) (or Eq. (9)), with the error about 15.8%, 25.3%, and 12.7% for N, H, and N-H, respectively. Details of the fit can be found in Table 3. The diffusion error is the sum of both statistical and fit contributions.

**Table 3:** Parameters given in the fits to the VACF in Eqs (7) and (9) that lead to diffusion coefficients as given in Eqs (2) and (3) for N, H and N-H at 1.9 g/cm^3^ and 4791 K.

Species	Method	*a*_0_(10^10^ cm^2^/s^2^)	*τ*_0_ (fs)	*a*_*i*_(10^10^ cm^2^/s^2^)	*τ*_*i*_ (fs)	*ω*_*i*_ (fs^−1^)	*α*_*i*_	*D*(10^−4^ cm^2^/s)
N	Eq. (7)	1.5 ± 0.05	11.7 ± 0.4	1.38	23.3 ± 0.3	0.09 ± 0.001	0.64	3.11 ± 0.1
N	Exp.	3.44 ± 0.07	10.45 ± 0.32					3.6 ± 0.2
H	Eq. (7)	−1.1 ± 0.15	27.1 ± 2.6	33.0	5.3 ± 0.1	0.21 ± 0.002	1.15	15.4 ± 0.3
				8.0	8.2 ± 0.2	0.52 ± 0.004	−0.05	
H	Exp.	47.16 ± 0.89	4.10 ± 0.11					19.3 ± 0.8
N-H	Eq. (9)	588.3 ± 20.5	6.8 ± 0.5	23.4	35.3 ± 12.2	0.23 ± 0.01	0.11	8.28 ± 1.1
				35.0	99.6 ± 21.2	0.16 ± 0.002	0.28	
				5.66	41.74 ± 5.3	0.08 ± 0.002	−1.59	
N-H	Exp.	716.5 ± 15.4	4.82 ± 0.16					9.33 ± 0.55

**Figure 6:**
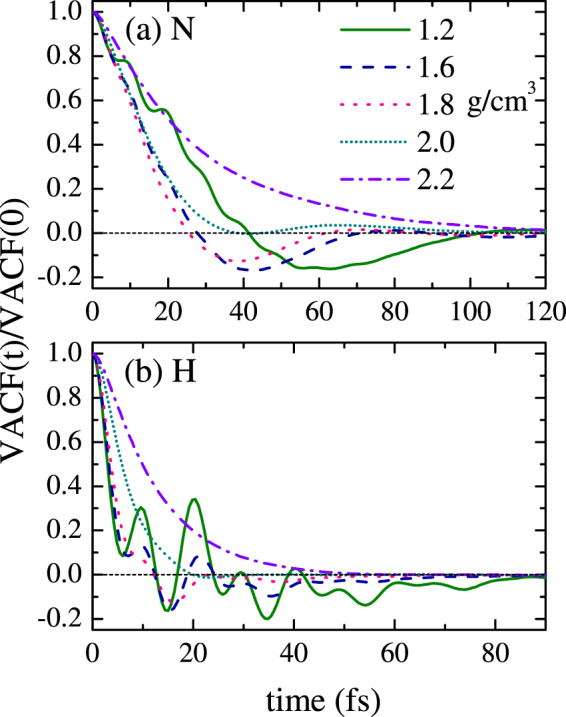
VACF for (**a**) N and (**b**) H for different densities along the principal hugoniot of ammonia.

Figure 6 presents the normalized VACF for N and H for different densities along the principal Hugoniot of ammonia. It is shown that the VACFs for N and H exhibit different structural forms and multiple time scales because of the mass difference. Evidently H diffuses on a shorter time scale. The VACF for N has less structures than H, which means N atoms are less correlated. As the density and temperature increase, both of the VACF for N and H evolve from complex structured form to simple exponential form, which corresponds to the process that the system changes from strong-coupling state to moderate-coupling state.

**Figure 7:**
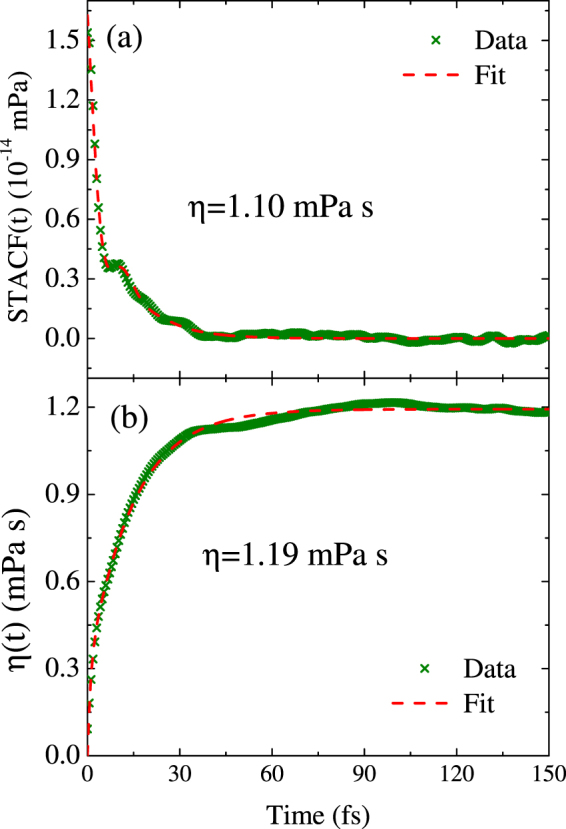
The STACF at 1.9 g/cm^3^ and 4791 K. (**a**) The STACF simulation data (green “x”) and the fit using Eq. (11). (**b**) The numerically integrated data with the fit to the analytic integral of Eq. (12). Here we set *β*_*i*_ = 1.

For the complex mixture, we use the functional of Eq. (11) to fit the stress-stress tensor autocorrelation function (STACF) and its integral for extracting viscosity. In Fig. 7 we show the STACF and its integrated value for the ammonia at 1.9 g/cm^3^ and 4791 K. In the fitting all of *β*_*i*_ are set to be unity. The fitting line goes through the data smoothly, and decreases to zero beyond 50 fs. Therefore the integrated value in a smaller time window could give the final viscosity value. We find that the fit for the STACF and its analytic integral yield results of *η* = 1.10 ± 0.21 mPa s and *η* = 1.19 ± 0.13 mPa s, respectively, which are in statistical agreement with each other. The longer fluctuations of the STACF result in the larger error bar in fitting. In addition, the STACF show evident structure with an increase about 7.8 fs, which could not be well fitted with a single exponential functional.

## Discussion

**Figure 8:**
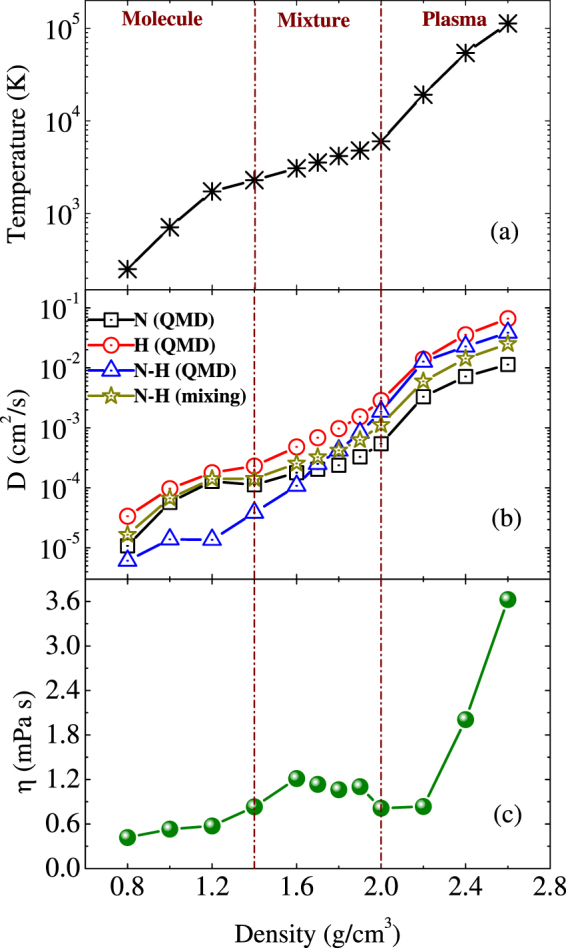
(**a**) Temperature, and (**b**) self- and mutual-diffusion coefficients, and (**c**) viscosity of ammonia along the principal Hugoniot. The mutual diffusion coefficients denoted by dark yellow stars are calculated by Eq. (5). The initial conditions for the principal Hugoniot are 0.6933 g/cm^3^ and 230 K.

Figure 8 presents the diffusion coefficient and viscosity as functions of density along the principal Hugoniot. For comparison, we replot the principal Hugoniot of temperature as functions of density in Fig. 8(a). As can be seen from Fig. 8, similar as the principal Hugoniots, both the diffusion coefficients and viscosity fall into three segments with their respective features, in accordance with the three structural regions respectively. In the molecular region, the hydrogen atoms have essentially the same diffusivity as the nitrogen atoms and the mutual diffusivity is much lower. In the mixture region, nitrogen atoms have diffusivity coefficients typical of fluids (~1.0 × 10^4^ cm^2^ s^−1^). In the plasma phase, the hydrogen atoms diffuse at least fourfold as fast as nitrogen. In addition, we note that in the plasma regime the mutual diffusion coefficients calculated using Eq. (5) in terms of self-diffusion coefficient agree well with those from QMD simulations directly. This means that the interactions between nitrogen and hydrogen atoms are very weak.

The complex behavior of viscosity is a consequence of the subtle interplay between coupling and kinetic effects. In kinetic regime with small coupling, the mechanism of bodily movement of particle is predominant and, as in a gas, the viscosity rises with temperature and density, reaching a maximum at about 1.6 g/cm^3^ and 3562 K, separating the molecular and mixture regimes. At intermediate coupling, the viscosity reaches a deep minimum at about 2.0 g/cm^3^ and 6036 K, which results from the similar contribution from the two competitive mechanisms. With the system becoming strongly coupled, the viscosity of ammonia behaves like dense ordinary liquids, demonstrating the importance of caging effect in this region.

**Figure 9:**
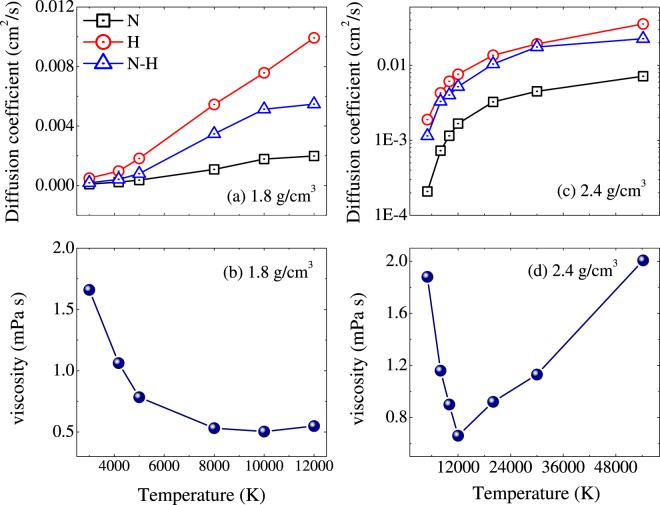
The diffusion and viscosity properties as functions of temperature along two isochores: 1.8 and 2.4 g/cm^3^.

Figure 9 shows the diffusion and viscosity properties as functions of temperature along two isochores: 1.8 and 2.4 g/cm^3^. In the temperature ranges we consider here, the thermodynamic factors computed with average-atom model almost keep constants of 1.289 and 1.334 for the isochores of 1.8 and 2.4 g/cm^3^, respectively. For these two cases, both self- and mutual diffusion coefficients increase systematically with temperature, except that the nitrogen atoms diffuse with typical values of fluid (~1.0 × 10^4^ cm^2^ s^−1^) for temperature below 5000 K at 1.8 g/cm^3^. In addition, along the isochore of 2.4 g/cm^3^, the self-diffusion coefficients for H are of the same order of magnitude as the mutual-diffusion coefficient, while the self-diffusion coefficients for N are reduced by almost one order of magnitude. The viscosity behaves much different for these two densities: At 1.8 g/cm^3^ the viscosity decreases with temperature, while at 2.4 g/cm^3^ the viscosity presents a shallow minimum as temperature increased to 12000 K. That is because the system lye in strong coupling (50 ≤ Γ) and intermediate coupling (10 ≤ Γ ≤ 50) regions at 1.8 and 2.4 g/cm^3^, respectively. As mentioned above, at intermediate coupling the two mechanisms of coupling and kinetic effects contribute with a similar magnitude, and thus leads to a minimum. At stronger coupling the caging effects dominates, and the viscosity exhibits the characterization of liquids.

In summary, we explore the complex behavior of ammonia at extreme conditions. The PDF and BACF suggest that ammonia undergoes three structural changes along principal Hugoniot: pure molecules, mixture, and plasma. This quantitatively demonstrates that profound chemistry process occurs in shocked ammonia. Especially, we note that the shocked nitrogen atoms form the chains with the dissociation of ammonia. Through using a complex fitting functional including structures and multiple time scales, accurate diffusion and viscosity constants of strong correlated ammonia are derived. It is found that the diffusion and viscosity exhibit distinct features in these three structural regions, which result from the competitive mechanisms of coupling and kinetic effects.

## Methods

The equilibrium configurations of the ions at different density-temperature states are determined by employing the quantum molecular dynamics (QMD) simulations, which are implemented in the Vienna *ab initio* simulation package (VASP)^18,19^. The equations of motion for active electrons are solved in the framework of finite-temperature density functional theory (FT-DFT)^20,21^, with the electronic states populated according to the Fermi-Dirac statistics. The ions propagate according to classical Newton equation, with the driven force derived via Hellmann-Feynman theorem at each MD step. The all-electron projector augmented wave (PAW) potentials are used to describe the interactions between the active electrons and the ions, with the exchange-correlation energy treated by the Perdew-Wang 91 parametrization of the generalized gradient approximation (GGA)^22–25^. The cutoff radiuses of the PAW psedopotential are 1.2 bohrs and 1.1 bohrs for N and H, respectively. Furthermore to gaurantee the reliability of the PAW potential, we have also chosen a PAW pseudopotential with shorter cutoff radius of rcut = 1.1 bohrs (N)/0.8 bohrs (H) to calculate the pressure and internal energy at the highest hugoniot point (2.6 g/cm^3^, 112663 K) for comparison. The electronic pressures are calculated to be 690.67 GPa and 693.38 GPa for the rcut = 1.2 bohrs (N)/1.1 bohrs (H) PAW potential and rcut = 1.1 bohrs (N)/0.8 bohrs (H) PAW potential, respectively, while the internal energies are 558.17 kJ/molecule and 559.14 kJ/molecule, respectively. Both of them are converged to be less than 0.5%, Thus the rcut = 1.2 bohrs (N)/1.1 bohrs (H) PAW potential is enough to reach convergence for the density ranges we considered in this manuscript. We also use the PBE^26^ and LDA^27^ functionals to study the influence of different exchange-correlation potentials on equation of state and structure, which are discussed in detail in the Results section.

In the simulations, the canonical (NVT) ensembles are set with the fixed number of particles, volume and temperature. The simulated density and temperature ranges from 0.8 to 2.6 g/cm^3^ and temperature from 250 to 120000 K, respectively. We fix the plane-wave cutoff at 1000 eV and use only the Γ point to sample the Brillouin zone of a cubic supercell with 256 atoms. A sufficient number of bands are included to ensure the occupation of the highest band to a level of 10^−5^. The simulations last 20000~40000 steps, with a time step varying for different density-temperature ranges: 0.5~1.0 fs for temperatures lower than 6000 K, 0.2~0.3 fs for higher temperature. These equilibrated trajectories provide a consistent set of structure and transport properties of ammonia.

To characterize the structural change in ammonia, we not only calculate the pair distribution function (PDF) for each possible bondings of N-N, H-H, and N-H regularly, but also analyze the MD trajectories through the use of the bond auto-correlation function (BACF), which can shed light on the complex chemistry occurring in ammonia under extreme pressure-temperature conditions. The BACF *β*(*t*) is defined as

1 $$\beta (t)=\frac{\langle \overrightarrow{B}\mathrm{(0)}\cdot \overrightarrow{B}(t)\rangle }{\langle \overrightarrow{B}\mathrm{(0)}\cdot \overrightarrow{B}\mathrm{(0)}\rangle },$$

where the bond vector $\vec{B}$ denotes a bit vector with value of 1 or 0 corresponding to the case for each bond of a certain type within or beyond a cutoff distance respectively. the direction of B vector is along the bond between two atoms. This correlation function describes the average duration of a bond between two atoms. In the present study, we use 1.7 Å for bonds between nitrogen atoms, 1.3 Å for bonds between nitrogen and hydrogen, and 0.9 Å for hydrogen-hydrogen bonds, which are the locations of the first minimum of pair distribution function for each species respectively. However, we note that the decay timescale (or the bond lifetime) is not strongly dependent on the chosen bond cutoff.

The transport properties, such as diffusivity and viscosity, are examined at different density-temperature conditions for ammonia. The self-diffusion coefficient of a particular ion species *D*_*α*_ is computed from the integral of the VACF^28,29^, which is

2 $${D}_{\alpha }=\frac{1}{3}{\int }_{0}^{\infty }\langle {{\bf{V}}}_{i}(t)\cdot {{\bf{V}}}_{i}\mathrm{(0)}\rangle \,{\rm{d}}t$$

with **V**_*i*_ being the velocity of the *i*th particle of species *α*. We also derive the mutual-diffusion coefficient *D*_*αβ*_ according to the Green-Kubo relation^30–32^

3 $${D}_{\alpha \beta }=\frac{Q}{3N{x}_{\alpha }{x}_{\beta }}{\int }_{0}^{\infty }\langle A(t)\,A\mathrm{(0)}\rangle \,{\rm{d}}t,$$

where

4 $$A(t)={x}_{\beta }\,\sum _{i=1}^{{N}_{\alpha }}{{\bf{V}}}_{i}(t)-{x}_{\alpha }\,\sum _{j=1}^{{N}_{\beta }}{{\bf{V}}}_{j}(t)\mathrm{.}$$

The thermodynamic factor *Q* accounts for nonideal mixing contributions to the Gibbs free energy with the expression of $Q={x}_{1}{x}_{2}{[{\partial }^{2}(\beta G/N)/\partial {x}_{1}^{2}]}_{P,T}$. This factor reduces to unity for ideal gas mixtures, but in ionic mixtures goes to (〈Z*〉 + 〈(Z*)^2^〉)/(〈Z*〉 + 〈Z*〉^2^) with Z* the average ionization degree^33^. *x*_*α*_ and *N*_*α*_ represent the concentration and particle number of species *α*, respectively. If the interspecies interactions remain small compared to the intraspecies ones, then the mutual diffusion coefficient takes a very simple form in terms of the concentrations and the self-diffusion coefficients:

5 $${D}_{\alpha \beta }={x}_{\beta }{D}_{\alpha }+{x}_{\alpha }{D}_{\beta }\mathrm{.}$$

This relation does not correspond to a standard mixing rule since the self-diffusion coefficients arise from simulations on the full mixture, not on each pure species.

In the stress-stress approach, the viscosity is computed from the STACF of non-diagonal components of the stress tensor (*P*_*xy*_, *P*_*yz*_, *P*_*zx*_, (*P*_*xx*_ − *P*_*yy*_)/2, and (*P*_*yy*_ − *P*_*zz*_)/2)^34,35^

$$\eta =\mathop{\mathrm{lim}}\limits_{t\to \infty }\,\overline{\eta }(t)$$

with

6 $$\overline{\eta }(t)=\frac{V}{{k}_{B}T}\,{\int }_{0}^{t}\,\langle {P}_{12}\mathrm{(0)}\,{P}_{12}(t^{\prime} )\rangle \,{\rm{d}}t^{\prime} .$$

It should be noted that to obtain similar statistical accuracy, the viscosity and mutual diffusion require much longer trajectories than the self-diffusion coefficient because the single-particle correlations are averaged over the particles and gain significant statistical improvement. To avoid the obstacle of computational cost, one can use empirical fits to the time dependence of the autocorrelation function, which can substantially shorten the length of the trajectory required. Then the long time behavior of the integrated functional form yields the desired transport property. The fitting functional form of simple exponential (or Gaussian) model is good enough to produce accurate results in the weakly coupling limit^36^. However, in strongly coupled regime, the autocorrelation function usually exhibits correlated and structured behavior, and thus the fitting functionals need more physics incorporated^37^. Especially for the dense mixture, there exist multiple restoring forces or multiple frequencies of collection motion acting on a given ion. Therefore, the fitting form with multiple times scales should be used to fit the autocorrelation function. As proposed in ref.^34^, the VACF could be given by

7 $$\langle {\bf{v}}(t)\cdot {\bf{v}}\mathrm{(0)}\rangle ={a}_{0}{e}^{-t/{\tau }_{0}}+\sum _{i=1}^{j}{a}_{i}{e}^{-t/{\tau }_{i}}[\cos \,({\omega }_{i}t)+{\alpha }_{i}\,\sin \,({\omega }_{i}t)],$$

where **v**(*t*) is the velocity at time *t*, *τ*_0_ and *τ*_*i*_ are decay times, and *ω*_*i*_ describes the frequency of collective motion (or restorative forces) near the onset of solidification, and *j* is the number of restoring-type forces included. Using Eq. (2) we immediately find the self-diffusion coefficient

8 $${D}_{\alpha }={a}_{0}{\tau }_{0}+\sum _{i=1}^{j}{a}_{i}{\tau }_{i}\frac{1+{\alpha }_{i}{\tau }_{i}{\omega }_{i}}{1+{\tau }_{i}^{2}{\omega }_{i}^{2}}\mathrm{.}$$

For the autocorrelation function of mutual-diffusion coefficient, the similar fitting functional form could be used as

9 $$\langle {\bf{A}}(t)\cdot {\bf{A}}\mathrm{(0)}\rangle ={B}_{0}{e}^{-t/{\tau }_{0}}+\sum _{i=1}^{j}{B}_{i}{e}^{-t/{\tau }_{i}}[\cos \,({\omega }_{i}t)+{\alpha }_{i}\,\sin \,({\omega }_{i}t)],$$

and the integrated result gives the mutual-diffusion coefficient

10 $${D}_{\alpha \beta }=\frac{Q}{N{x}_{\alpha }{x}_{\beta }}[{B}_{0}{\tau }_{0}+\sum _{i=1}^{j}{B}_{i}{\tau }_{i}\frac{1+{\alpha }_{i}{\tau }_{i}{\omega }_{i}}{1+{\tau }_{i}^{2}{\omega }_{i}^{2}}].$$

A multi-exponential fit is used to describe the STACF based on a Kohlrausch law

11 $$\langle {\bf{s}}(t)\cdot {\bf{s}}\mathrm{(0)}\rangle ={b}_{0}{e}^{{(-t/{\tau }_{0})}^{{\beta }_{0}}}+\sum _{i=1}^{j}{b}_{i}{e}^{{(-t/{\tau }_{i})}^{{\beta }_{i}}}\,\cos \,({\omega }_{1}t),$$

where **s**(*t*) is the stress-tensor at time *t*. Applying Eqs (6–11) yields the analytic expression of viscosity when *β*_*i*_ = 1:

12 $$\eta ={b}_{0}{\tau }_{0}+\sum _{i=1}^{j}\frac{{b}_{i}{\tau }_{i}}{1+{({\tau }_{i}{\omega }_{i})}^{2}}.$$

The statistical error inherent in computing correlation functions from molecular dynamics trajectories for Eq. (7) is

13 $$\varepsilon =\sqrt{\frac{2}{N{T}_{traj}}({c}_{0}{\tau }_{0}+\sum _{i=1}^{j}{c}_{i}{\tau }_{i}\frac{1+{\alpha }_{i}{\tau }_{i}{\omega }_{i}}{1+{\tau }_{i}^{2}{\omega }_{i}^{2}}}$$

with *c*_*i*_ = *a*_*i*_/Σ_*i*_ *a*_*i*_. The differences between the statistical error for Eqs (7) and (9) are that the disappearance of $\frac{1}{\sqrt{N}}$ and the expression of *c*_*i*_ changed to be *B*_*i*_/Σ_*i*_ *B*_*i*_. Similarly, for *β*_*i*_ = 1 the idealized error for Eq. (11) could be evaluated as

14 $$\varepsilon =\sqrt{\frac{2}{{T}_{traj}}({d}_{0}{\tau }_{0}+\sum _{i=1}^{j}\frac{{d}_{i}{\tau }_{i}}{1+{\tau }_{i}^{2}{\omega }_{i}^{2}}},$$

in which *d*_*i*_ = *b*_*i*_/Σ_*i*_ *b*_*i*_.
